# Aerobic Cytotoxicity of Aromatic *N-*Oxides: The Role of NAD(P)H:Quinone Oxidoreductase (NQO1)

**DOI:** 10.3390/ijms21228754

**Published:** 2020-11-19

**Authors:** Aušra Nemeikaitė-Čėnienė, Jonas Šarlauskas, Lina Misevičienė, Audronė Marozienė, Violeta Jonušienė, Mindaugas Lesanavičius, Narimantas Čėnas

**Affiliations:** 1 State Research Institute Center for Innovative Medicine, Santariškių St. 5, LT-08406 Vilnius, Lithuania; ausra.ceniene@imcentras.lt; 2 Institute of Biochemistry of Vilnius University, Saulėtekio 7, LT-10257 Vilnius, Lithuania; jonas.sarlauskas@bchi.vu.lt (J.Š.); lina.miseviciene@bchi.vu.lt (L.M.); audrone.maroziene@bchi.vu.lt (A.M.); mindaugas.lesanavicius@gmail.com (M.L.); 3 Institute of Biosciences of Vilnius University, Saulėtekio 7, LT-10257 Vilnius, Lithuania; violeta.jonusiene@gf.vu.lt

**Keywords:** tirapazamine, reductive activation, NAD(P)H:quinone oxidoreductase, oxidative stress, cytotoxicity

## Abstract

Derivatives of tirapazamine and other heteroaromatic *N-*oxides (ArN→O) exhibit tumoricidal, antibacterial, and antiprotozoal activities, which are typically attributed to bioreductive activation and free radical generation. In this work, we aimed to clarify the role of NAD(P)H:quinone oxidoreductase (NQO1) in ArN→O aerobic cytotoxicity. We synthesized 9 representatives of ArN→O with uncharacterized redox properties and examined their single-electron reduction by rat NADPH:cytochrome P-450 reductase (P-450R) and *Plasmodium falciparum* ferredoxin:NADP^+^ oxidoreductase (*Pf*FNR), and by rat NQO1. NQO1 catalyzed both redox cycling and the formation of stable reduction products of ArN→O. The reactivity of ArN→O in NQO1-catalyzed reactions did not correlate with the geometric average of their activity towards P-450R- and *Pf*FNR, which was taken for the parameter of their redox cycling efficacy. The cytotoxicity of compounds in murine hepatoma MH22a cells was decreased by antioxidants and the inhibitor of NQO1, dicoumarol. The multiparameter regression analysis of the data of this and a previous study (DOI: 10.3390/ijms20184602) shows that the cytotoxicity of ArN→O (*n =* 18) in MH22a and human colon carcinoma HCT-116 cells increases with the geometric average of their reactivity towards P-450R and *Pf*FNR, and with their reactivity towards NQO1. These data demonstrate that NQO1 is a potentially important target of action of heteroaromatic *N-*oxides.

## 1. Introduction

**Scheme 1:**
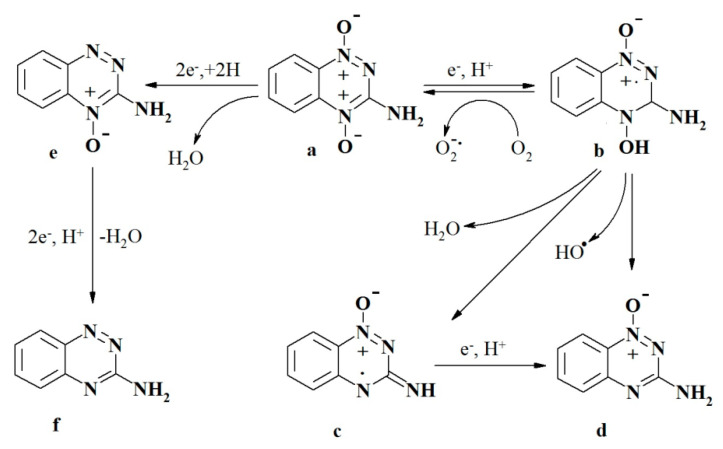
Pathways of the reduction of tirapazamine in the cell.

*N-*oxides of 1,2,4-benzotriazine, quinoxaline, and phenazine (ArN→O) possess promising antitumor, antiprotozoal, and antibacterial activities, including their potential application as hypoxia-specific antitumor agents ([1,2,3,4,5,6], and references therein). In most cases, their action is attributed to the enzymatic reduction and free radical generation. Among their representatives, the reactions of 3-amino-1,2,4-benzotriazine-1,4-dioxide (tirapazamine, TPZ) and its derivatives have been studied most comprehensively. TPZ (**a**) is enzymatically reduced in a single-electron way to a free radical (**b**), which forms DNA-damaging species under hypoxic conditions, namely, an oxidizing hydroxyl radical (OH^.^) ([2,7,8], and references therein), and/or a highly reactive benzotriazinyl radical (**c**) that abstracts a hydrogen atom from DNA ([9,10], and references therein (Scheme 1). The nature of DNA-damaging species remains a matter of debate. The final relatively nontoxic metabolites of TPZ are its mono-*N-*oxide (**d**)**,** formed possibly via free radical (**c**) intermediate, and its nor-oxide (**f**) [2,7,11]. Their formation is strongly inhibited under aerobic conditions.

The aerobic cytotoxicity of ArN→O is typically attributed to their redox cycling (Scheme 1), leading to the formation of superoxide (O_2_^−^) and subsequent oxidative stress [1,2]. This phenomenon deserves certain interest because some TPZ analogs possess anticancer activity at micromolar concentrations even under oxic conditions [12,13,14], and/or are toxic to normal tissues [15]. Besides, the oxidative stress-type mammalian cell cytotoxicity may be important as a side effect in the antimicrobial and antiparasitic action of ArN→O ([2,6], and references therein). Flavoenzyme NADPH:cytochrome P-450 reductase (P-450R) and/or insufficiently characterized intranuclear NAD(P)H-oxidizing flavoenzymes are assumed to be mainly responsible for the single-electron reduction of ArN→O irrespectively of hypoxic or oxic conditions ([16,17], and references therein). Another enzyme, potentially relevant to the cytotoxicity of ArN→O, is NAD(P)H:quinone oxidoreductase (NQO1, DT-diaphorase). NQO1 is a dimeric enzyme containing one molecule of FAD per subunit, located mainly in the cytosol [18]. It reduces quinones and nitroaromatic compounds in an obligatory two-electron way ([19,20], and references therein), and is considered to be a target of bioreductively-activated quinone antitumor agents [21]. NQO1 reduces TPZ and its derivatives much slower than quinones in a mixed single- and the two-electron way [22]. The data on the impact of NQO1 on cytotoxicity of TPZ are controversial [22,23,24,25]. However, the studies of its reactions with ArN→O deserve certain interest because the activity of NQO1 is frequently elevated in various tumors ([26], and references therein).

The aerobic cytotoxicity of several ArN→O in MH22a mouse hepatoma cells roughly increased with their single-electron reduction midpoint potential (*E*^1^_7_) [22], which demonstrates an involvement of P-450R and/or other single-electron transferring flavoenzymes. In parallel, the cytotoxicity was decreased by dicoumarol, which points to the possible participation of NQO1 in cytotoxic events. In this work, we extended these studies using a series of previously unexplored aromatic *N-*oxides and have shown that NQO1 unequivocally contributes to the cytotoxicity of ArN→O.

## 2. Results

### 2.1. Reactions of Aromatic N-Oxides with Flavoenzymes Dehydrogenases-Electrontransferases

**Figure 1:**
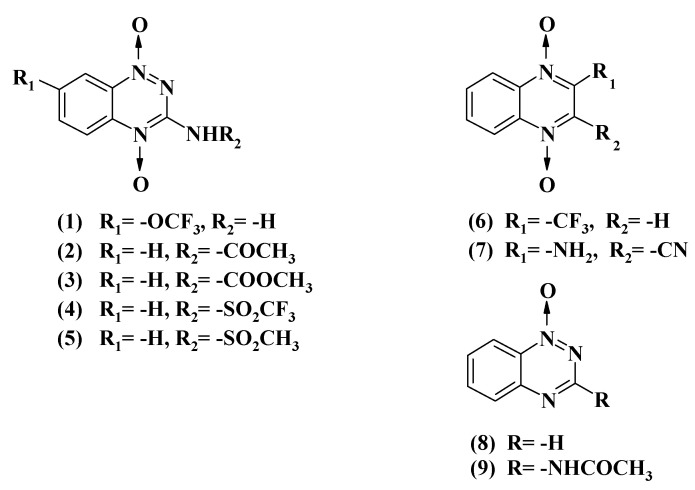
Formulae of heteroaromatic *N*-oxides used in this work: derivatives of tirapazamine (**1**–**5**), quinoxaline-1,4-dioxide (**6**,**7**), and 1,2,4-benzotriazine-1-oxide (**8**,**9**).

Previously we studied the enzymatic reactions and cytotoxicity of a number of ArN→O, mostly 7-substituted tirapazamine derivatives, whose *E*^1^_7_ varied between −0.318 V and −0.575 V [22]. In this work, we extended these studies using representatives of several groups of ArN→O with uncharacterized values of *E*^1^_7_ (Figure 1).

Table 1 contains bimolecular rate constants (*k*_cat_/*K*_m_) of reduction of examined ArN→O by P-450R and *Plasmodium falciparum* ferredoxin:NADP^+^ oxidoreductase (*Pf*FNR), which was used as a model single-electron transferring enzyme [27]. For the most active oxidants of P-450R studied in this work, 7-CF_3_O-tirapazamine, and 3-CH_3_CONH- and 3-CH_3_OCONH-substituted 1,2,4-benzotriazine-1,4-dioxides (Table 1), the *k*_cat_ at their saturated concentrations were in the range of 16.0–18.0 s^−1^, i.e., close to 50% of the rate of cytochrome *c* reduction by P-450R. The above compounds were also the most active oxidants of *Pf*FNR (Table 1), with *k*_cat_ of their reduction reaching 11.0–13.0 s^−1^. In other cases, the reaction rates were proportional to the concentration of compounds up to the limits of their solubility, 300–600 µM. Because *Pf*FNR does not reduce cytochrome *c* directly [27], it was possible to demonstrate the redox cycling of synthesized ArN→O monitoring the reduction of cytochrome *c* added to the reaction mixture. The rates of this reaction were equal to 175–190% NADPH oxidation rate. The cytochrome *c* reduction was inhibited by 20–40% by 100 U/mL superoxide dismutase (SOD), which points to the formation of superoxide.

Typically, the logarithms of *k*_cat_/*K*_m_ of single-electron reduction of ArN→O by flavoenzymes dehydrogenases-electrontransferases such as P-450R or *Pf*FNR linearly increase with *E*^1^_7_ [22,27]. This is attributed to an “outer-sphere” electron transfer mechanism, where the reactivity of compounds is insignificantly influenced by their structural peculiarities [28]. Therefore, log *k*_cat_/*K*_m_ of homologous oxidants in single-electron enzymatic reduction reaction may serve as the parameter characterizing their *E*^1^_7_, i.e., the energetic of single-electron reduction. The use of the geometric average of *k*_cat_/*K*_m_ obtained in several enzymatic systems improves the prediction accuracy [29,30]. Table 1 contains the logarithms of geometric averages of *k*_cat_/*K*_m_ of ArN→O in P-450R- and *Pf*FNR-catalyzed reactions (log *k*_cat_/*K*_m_ (avge) = 0.5 log *k*_cat_/*K*_m_ (P-450R) + 0.5 log *k*_cat_/*K*_m_ (*Pf*FNR)). For comparison, the reduction rate constants of compounds **10**–**18** obtained in our previous studies [22,27] are also presented (Table 1). The log *k*_cat_/*K*_m_ (avge) of these compounds correlates well with their *E*^1^_7_ values:

log *k*_cat_/*K*_m_ (avge) = (7.23 ± 0.29) + (6.96 ± 0.65) *E*^1^_7_, *r*^2^ = 0.9430. (1)

A similar correlation is obtained including the assumed *E*^1^_7_ values for compounds **6**,**8** (Table 1):

log kcat/Km (avge) = (7.24 ± 0.27) + (6.97 ± 0.59) E17, r2 = 0.9400.(2)

For compounds with unavailable *E*^1^_7_ values, the introduction of electron-accepting substituents into 3-NH_2_ group or into 7- or 2-positions of the aromatic system in most cases increased their log *k*_cat_/*K*_m_ (avge) as compared with parent compounds (Table 1). Therefore, in this case, log *k*_cat_/*K*_m_ (avge) appears to be a suitable parameter describing the ease of single-electron reduction of ArN→O.

**Table 1:** The single-electron reduction midpoint potentials (*E*^1^_7_) of aromatic *N-*oxides, the steady-state bimolecular rate constants (*k*_cat_/*K*_m_) of their reduction by P-450R and *Pf*FNR, and the logs of the geometric averages of their reactivity (log *k*_cat_/*K*_m_ (avge)).

No.	Compound	*E*^1^_7_ (V) ^a^	*k*_cat_/*K*_m_ (M^−1^·s^−1^)		log *k*_cat_/*K*_m_ (avge)
			P-450R	*Pf*FNR	
1	7-CF_3_O-tirapazamine		4.6 ± 0.4 × 10^4^	3.8 ± 0.4 × 10^4^	4.62
2	3-CH_3_CONH-1,2,4-benzotriazine-1,4-dioxide		7.0 ± 0.5 × 10^4^	6.2 ± 0.5 × 10^4^	4.82
3	3-CH_3_OCONH-1,2,4-benzotriazine-1,4-dioxide		8.0 ± 0.9 × 10^4^	4.8 ± 0.4 × 10^4^	4.78
4	3-CF_3_SO_2_NH-1,2,4-benzotriazine-1,4-dioxide		2.5 ± 0.3 × 10^4^	2.6 ± 0.3 × 10^4^	4.41
5	3-CH_3_SO_2_NH-1,2,4-benzotriazine-1,4-dioxide		2.7 ± 0.3 × 10^3^	7.9 ± 0.5 × 10^3^	3.67
6	2-CF_3_-quinoxaline-1,4-dioxide	(−0.465) ^b^	2.7 ± 0.3 × 10^4^	8.9 ± 0.7 × 10^3^	4.19
7	2-NH_2_-3-CN-quinoxaline-1,4-dioxide		4.7 ± 0.4 × 10^3^	1.8 ± 0.2 × 10^4^	3.96
8	1,2,4-Benzotriazine-1-oxide	(−0.431) ^b^	1.7 ± 0.2 × 10^4^	4.3 ± 0.3 × 10^3^	3.94
9	3-CH_3_CONH-1,2,4-benzotriazine-1-oxide		8.7 ± 0.9 × 10^3^	1.6 ± 0.1 × 10^3^	3.58
ArN→O with available *E*^1^_7_ values					
10	1,2,4-Benzotriazine-1,4-dioxide	−0.318	4.3 ± 0.4 × 10^5 c^	2.5 ± 0.3 × 10^4^	5.00
11	7-CF_3_-tirapazamine	−0.345	8.7 ± 0.7 × 10^4 c^	5.2 ± 0.4 × 10^4 d^	4.83
12	7-Cl-tirapazamine	−0.400	6.9 ± 0.7 × 10^4 c^	3.7 ± 0.4 × 10^4 d^	4.71
13	7-F-tirapazamine	−0.400	3.4 ± 0.3 × 10^4 c^	2.7 ± 0.2 × 10^4 d^	4.48
14	Tirapazamine	−0.455	1.1 ± 0.1 × 10^4 c^	4.4 ± 0.5 × 10^3 d^	3.84
15	7-CH_3_-tirapazamine	−0.474	8.6 ± 0.7 × 10^3 c^	5.0 ± 0.6 × 10^3 d^	3.82
16	7-C_2_H_5_O-tirapazamine	−0.494	4.5 ± 0.5 × 10^3 c^	4.5 ± 0.5 × 10^3 d^	3.65
17	3-Amino-1,2,4-benzotriazine-1-oxide	−0.568	2.8 ± 0.2 × 10^3 c^	3.2 ± 0.2 × 10^3 d^	3.48
18	Quinoxaline-1,4-dioxide	−0.575	3.3 ± 0.2 × 10^3 c^	8.2 ± 0.9 × 10^2 d^	3.22

^a^*E*^1^_7_ of compounds taken from Ref. [7,31,32], ^b^ Calculated using the *E*^1^_7_ differences between compounds **11** and **14**, 0.110 V, and compounds **17** and **14**, −0.113 V, respectively, ^c^ Taken from Ref. [22], ^d^ Taken from Ref. [27].

### 2.2. NQO1-Catalyzed Reduction of Aromatic N-Oxides

NQO1 performs a slow reduction of ArN→O [22,33]. The reactivity of compounds **1**–**9** determined in this work was similar to that of compounds **10**–**18** [22], except for its larger variation (Table 2). The log *k*_cat_/*K*_m_ for compounds **1**–**18** (Table 2) does not correlate with the energetics of their single-electron reduction, because of its dependence on *E*^1^_7_ or log *k*_cat_/*K*_m_ (avge) is described by *r*^2^ = 0.0451 and *r*^2^ = 0.1441, respectively. Because the reactivity of quinones towards NQO1 depends on their van der Waals volume (VdWvol) [19], we also used this parameter for calculations (Table 2). However, the combined use of log *k*_cat_/*K*_m_ (avge) and VdWvol or VdWvol + (VdWvol)^2^ as independent variables also did not give satisfactory results, being described by *r*^2^ = 0.2302 and *r*^2^ = 0.3846, respectively. Thus, the structural requirements for the fast reduction of ArN→O by NQO1 remain unclear at this time.

**Figure 2:**
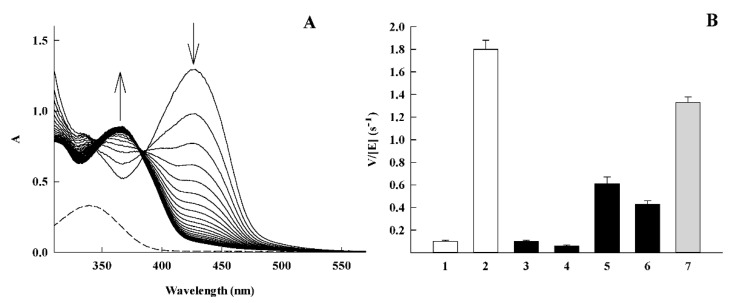
Reduction of 3-CH_3_CONH-1,2,4-benzotriazine-1,4-dioxide (**2**) by NAD(P)H:quinone oxidoreductase (NQO1). (**A**) The spectral changes of 200 µM (**2**) in the presence of 50 nM NQO1, and 50 µM NADPH and NADPH regeneration system. The absorbance of NADPH is shown by a dashed line. The scans are recorded each 15 min. (**B**) The rates of NQO1-catalyzed oxidation of NADPH (1,2), reduction of cytochrome *c* (3–6), and depletion of 200 µM (**2**) in the presence of 200 µM NADPH (7). Additions: NADPH (1), NADPH + (**2**) (2,7), NADPH + cytochrome *c* (3), NADPH + cytochrome *c* + 100 U/mL SOD (4), NADPH + (**2**) + cytochrome *c* (5), NADPH + (**2**) + cytochrome *c* + SOD (6), *n* = 3, *p* < 0.01 for 3 against 4 and for 5 against 6.

Under anaerobic conditions, NQO1 reduces TPZ into its 4-monoxide (**d**) and nor-oxide (**f**) (Scheme 1) [33]. The reduction is strongly inhibited by O_2_ [33] and is accompanied by redox cycling process [22]. Because of the low rate of this reaction, we selected more reactive 3-CH_3_CONH-1,2,4-benzotriazine-1,4-dioxide (**2**) (Table 2) for subsequent studies. The temperature dependence of *k*_cat_*/K*_m_ of reaction determined according to NADPH oxidation in the absence of activators gives the activation enthalpy (∆H^≠^) of 37.76 ± 1.93 kJ mol^−1^ and the activation entropy (∆S^≠^) of −42.97 ± 6.68 J mol^−1^ K^−1^. In the presence of the NADPH regeneration system under aerobic conditions, the disappearance of compound absorbance at 427 nm is accompanied by absorbance rise at 375 nm (Figure 2A). This shows that the possible reaction product is CH_3_CONH-1,2,4-benzotriazine-1-oxide (**9**) (Figure 1), which possesses absorbance maximum at this wavelength (data not shown). The more detailed characterization of reaction products is beyond the scope of the present work.

The data of Figure 2B show that the rate of 3-CH_3_CONH-1,2,4-benzotriazine-1,4-dioxide disappearance was equal to 70–75% NADPH oxidation rate determined in the absence of NADPH regeneration system. The rate of compound disappearance was directly proportional to NQO1 concentration in the range of 12.5–100 nM. The reaction was accompanied by the reduction of added cytochrome *c* with the rate being equal to 30–35% NADPH oxidation rate (Figure 2B). The reduction of cytochrome *c* was partly inhibited by superoxide dismutase, which demonstrates the formation of O_2_^−^. During the reduction of (**2**) by *Pf*FNR, the rate of added cytochrome *c* reduction was much higher, being equal to 180–190% NADPH oxidation rate. The cytochrome *c* reduction was inhibited by SOD by 20%.

### 2.3. Cytotoxicity of Aromatic N-Oxides

In cytotoxicity studies, we determined the concentrations of ArN→O for 50% cell survival (cL_50_) in murine hepatoma MH22a cells and their concentrations for 50% of maximal inhibition (GI_50_) of the proliferation of human colon adenocarcinoma HCT-116 cells (Table 3). The data of our previous study [22] are also presented for comparison.

We previously demonstrated the prooxidant character of cytotoxicity of ArN→O in MH22a cells [22]. In line with this, the cytotoxicity of several randomly selected ArN→O explored in this work, was decreased by desferrioxamine (DESF) and the antioxidant *N,N′-*diphenyl-*p-*phenylenediamine (DPPD), and enhanced by 1,3-bis(2-chloroethyl)-1-nitrosourea (BCNU), the latter inactivating glutathione reductase and depleting reduced glutathione [35] (Table 4). Besides, their cytotoxicity was decreased by an inhibitor of NQO1, dicoumarol (DIC) (Table 4).

In quantitative analysis of cytotoxicity of ArN→O, we used the ease of their single-electron reduction (log *k*_cat_/*K*_m_ (avge), Table 1), lipophilicity (log *D,* Table 3)), and reactivity towards NQO1 (log *k*_cat_/*K*_m_ (NQO1), Table 2) as the correlation parameters. The data were analyzed using the linear multiparameter regression:

log cL_50_ (or log GI_50_) = *a* + *b* log *k*_cat_/*K*_m_ (avge) + *c* log *D* + *d* log *k*_cat_/*K*_m_ (NQO1) (3)

The calculated coefficients in Equation (3) (Table 5) show that the cytotoxicity of compounds in both cell lines roughly increases with their log *k*_cat_/*K*_m_ (avge) when it is used as a single variable. The introduction of log *k*_cat_/*K*_m_ (NQO1) as a second variable improved the correlations, and also unequivocally demonstrated that the ArN→O cytotoxicity increases with their reactivity towards NQO1 (Table 5).

In all cases (Table 5), the coefficient *d* characterizing an involvement of NQO1 (Equation (3)) is statistically significant (*p* < 0.05). The use of log *D* as an additional variable did not improve the correlations and demonstrated a limited impact of lipophilicity on ArN→O cytotoxicity (Table 5), which is similar to the data of a previous study [31].

The role of NQO1 in the cytotoxicity of ArN→O was additionally assessed using bovine leukemia virus-transformed lamb embryo kidney fibroblasts (line FLK [36]), murine embryonic liver cells (line BNL CL.2 [37]), and primary mice splenocytes [38] (Supplemental Information, Table S1). In this case, DIC protected against the cytotoxicity of TPZ and 3-CH_3_CONH-1,2,4-benzotriazine-1,4-dioxide in cell lines with a relatively high content of NQO1, but did not protect against their cytotoxicity in mice splenocytes with low NQO1 content, 4.0 nmol cytochrome *c* reduced × min^−1^ × mg^−1^ [38] (Table S2).

## 3. Discussion

In this study, we examined the relationship between enzymatic redox properties and aerobic cytotoxicity of aromatic *N*-oxides with a particular emphasis on the role of NQO1. The rate constants of single-electron reduction of ArN→O **1–9** by P-450R and *Pf*FNR (Table 1) are in line with previous findings [22,27], showing that the reactivity is mostly determined by the electron-accepting potency of ArN→O, and hardly depend on their structural peculiarities. Their log *k*_cat_/*K*_m_ (avge) proved to be a reasonable substitute for *E*^1^_7_ because of their good interrelationship (Equations (1) and (2)). Interestingly, its use for the description of ArN→O cytotoxicity (Equation (3), Table 5) resulted in *r*^2^ values close to those in previously obtained log (cytotoxicity) vs. *E*^1^_7_ relationships [31]. On the other hand, the use of a large group of ArN→O with highly variable and reduction potential-independent reactivity towards NQO1 (Table 2), enabled us to characterize the role of this enzyme in their cytotoxicity.

In regards to the possible mechanism of reduction of ArN→O by NQO1, one may note their structural similarity with 1,4-naphthoquinones, which are planar bicyclic molecules with similar electron self-exchange rate constants, ~10^8^ M^−1^ s^−1^ [22,39]. The available data on the mechanism of quinone reduction are summarized below: (i) a net two-electron reduction of quinones by NQO1 is attributed to low stability of its FAD semiquinone (FAD^−^), 8% under equilibrium [40], and the efficient electronic coupling, which is provided by the sandwiching of quinone ring between isoalloxazine ring of FAD and Phe-178’ [41]; (ii) The instability of FAD^−^ does not restrict the reactivity of NQO1 with single-electron acceptors [19]. NQO1 reduces nitroaromatic compound tetryl in a mixed single- and two-electron way [42], and its reactions with quinones may be described by a multistep (e^−^,H^+^,e^−^) hydride transfer model [19]; (iii) The reactivity of quinones roughly increases with their E17 and sharply decreases when their VdWvol is above 200 Å^3^ [19]. An increase in VdWvol may increase the distance between N5 of isoalloxazine and quinone ring [43], and (iv) The ΔS^≠^ values of ‘fast’ quinone oxidants with VdWvol ≤ 200 Å^3^ are lower than ΔS^≠^ of ‘slow’ quinones (VdWvol > 200 Å^3^) by 40–70 J mol^−1^ K^−1^ [19]. More negative ΔS^≠^ values point to the stronger electronic coupling between the molecules of reactants [44].

In our case, NQO1 reduces ArN→O 10^2^–10^3^ times slower than 1,4-naphthoquinones with comparable *E*^1^_7_ and VdWvol values, −0.36–−0.46 V, and 180–200 Å^3^, respectively [19]. The use of a relatively fast NQO1 substrate, 3-CH_3_CONH-1,2,4-benzotriazine-1,4-dioxide (**2**) (Table 2, Figure 2A,B), enabled the characterization of this process more thoroughly. The SOD-sensitive reduction of added cytochrome *c* during the reaction (Figure 2B) points to the formation of a free radical ((**b**)**,** Scheme 1), which enters the redox equilibrium with O_2_/O_2_^−^ couple. In our opinion, the lower rate of ArN→O reduction when compared to quinones and the dissociation of free radicals may be caused by a weak electronic coupling of reactants. The electronic coupling may be weakened by the loss of planarity of (**b**) due to the adoption of markedly pyramidal geometries of N1 and N4 in a free radical state [45]. This is supported by ∆S^≠^ value of 3-CH_3_CONH-1,2,4-benzotriazine-1,4-dioxide, -43 J mol^−1^ K^−1^, which is far less negative than ΔS^≠^ of ‘fast’ quinone oxidants, −85–−75 J mol^−1^ K^−1^ [19]. However, the yield of free radicals in NQO1-catalyzed reduction of this compound, expressed as the ratio of cytochrome *c* reduction and doubled NADPH oxidation rates [27], is close to 30% (Figure 2B). This is much lower than the single-electron flux of 90–95%, observed in the reaction with single-electron transferring *Pf*FNR. In parallel, 3-CH_3_CONH-1,2,4-benzotriazine-1,4-dioxide disappears in the course of the reaction (Figure 2A,B). Analogous although relatively slower process takes place during the reduction of TPZ by NQO1 [22]. The sum of this reaction rate and the halved rate of cytochrome *c* reduction is close to the total rate of NADPH oxidation (Figure 2B). The disappearance of oxidant is not caused by free radical dismutation, because its rate exhibits a linear, but not a square dependence on the enzyme concentration that is characteristic for the dismutation-driven process [46]. Thus, the possible explanation is that NQO1 performs mixed single- and two-electron reduction of ArN→O with the initial single-electron transfer step. The elucidation of the mechanism of a second electron transfer is more problematic because of debates whether the 1,4-dioxide (**a**) reduction product, 1-monoxide (**d**), is formed from free radical (**b**) directly, or via aryl radical (**c**) (Scheme 1) [2,7,8,9,10,11]. This problem is an object of our future studies, together with the detailed characterization of the reaction product(s).

The multiparameter regression analysis (Equation (3), Table 3 and Table 5) provides the first quantitative confirmation of NQO1 contribution to the cytotoxicity of ArN→O in two cell lines. Given the data currently available, the most credible mechanism of their action is NQO1-catalyzed redox cycling. Apart from MH22a cells (Table 4), dicoumarol protected against TPZ aerobic cytotoxicity in A549 cells, besides, the TPZ-resistant A549 subline almost completely lost NQO1 activity [24]. On the other hand, there also exist data on the absence of the relationship between the content of NQO1 and the cytotoxicity of TPZ in several cell lines [23,25]. Because P-450R and/or nuclear reductases typically play the most significant role in the bioactivation of TPZ [16,17], it is possible that in certain cases their action may shield the role of NQO1. One may also expect that the role of NQO1 may be more expressed using its substrates more efficient than TPZ and especially possessing lower *E*^1^_7_ values, i.e., lower reactivity towards P-450R (Table 1 and Table 2). In conclusion, our data indicate that NQO1 may be an important target of ArN→O in the cell. The further studies of electron transfer mechanism and substrate specificity of these reactions may increase the variety of efficient therapeutic agents of this group, and contribute to the optimization of their action.

## 4. Materials and Methods

### 4.1. Enzymes and Chemicals

Recombinant rat P-450R was a generous gift of Dr. Alexey Yantsevich (Institute of Bioorganic Chemistry, NAS of Belarus, Minsk, Belarus), and was prepared as described in [47]. Recombinant *P. falciparum* ferredoxin:NADP^+^ oxidoreductase (*Pf*FNR) was a generous gift of Professor Alessandro Aliverti (Department of Biosciences, Universita degli Studi di Milano, Milano, Italy), and was prepared as previously described in [48]. NQO1 was prepared from rat liver according to Prochaska [49]. The enzyme concentrations were determined spectrophotometrically according to ε_456_ = 21.4 mM^−1^·cm^−1^ (P-450R [47]), *ε*_454_ = 10.0 mM^−1.^cm^−1^ (PfFNR [48]), and ε_460_ = 11.0 mM^−1^·cm^−1^ (NQO1 [49]). Derivatives of tirapazamine (**1–5**) (Figure 1) were synthesized as described in [31,50]. Quinoxaline-1,4-dioxide and its derivatives (**6**,**7**) (Figure 1) were synthesized as described in [51,52]. 1,2,4-Benzotriazine-1,4-dioxide and derivatives of 1,2,4-benzotriazine-1-oxide (**8**,**9**) (Figure 1) were synthesized as described in [53,54]. The compound purity was characterized by IR and NMR spectrometry, melting point, and elemental analysis. NADPH, cytochrome *c*, superoxide dismutase, and other reagents were obtained from Sigma-Aldrich (St. Louis, MO, USA), and used as received.

### 4.2. Enzymatic Assays

The kinetic measurements were carried out spectrophotometrically using a PerkinElmer Lambda 25 spectrophotometer (PerkinElmer, Waltham, MA, USA) in 0.1 M K-phosphate buffer (pH 7.0) containing 1 mM EDTA at 25 °C. The enzyme activities determined according to the rate of reduction of 50 µM cytochrome *c* (∆ε_550_ = 20 mM^−1^·cm^−1^) at substrate concentrations indicated below were close to those reported previously [22]: 39 s^−1^ (P-450R, [NADPH] = 100 µM), and 1750 s^−1^ (NQO1, [NADPH] = 150 µM, [menadione] = 10 µM). In this case, 0.01% Tween 20 and 0.25 mg/mL bovine serum albumin were added as NQO1 activators. The activity of *Pf*FNR, determined according to the reduction rate of 1.0 mM ferricyanide (∆ε_420_ = 1.03 mM^−1.^cm^−1^) at [NADPH] = 100 µM was equal to 48 s^−1^. The initial rates of P-450R- or *Pf*FNR-catalyzed NADPH-dependent *N-*oxide reduction were determined according to ∆ε_340_ = 6.2 mM^−1^·cm^−1^ after the subtraction of intrinsic NADPH oxidase activities of enzymes, 0.05 s^−1^ (P-450R), and 0.1 s^−1^ (*Pf*FNR). The stock solutions of oxidants were prepared in DMSO (dilution factor 100). The initial rates of NQO1-catalyzed ArN→O reduction were determined according to the rate of NADPH oxidation. In this case, the rates were corrected for 340 nm absorbance changes due to ArN→O disappearance. The latter were obtained under the same conditions in the presence of NADPH regeneration system, 10 mM glucose-6-phosphate and 0.3 mg/mL glucose-6-phosphate dehydrogenase. The loss of 3-CH_3_CONH-1,2,4-benzotriazine-1,4-dioxide during its reduction by NQO1 was monitored according to ∆ε_427_ = 6.45 mM^−1.^cm^−1^, which was calculated according to the absorbance difference of compounds (**2**) and (**9**) (Figure 1). The values of turnover rate, *k*_cat_, reflecting the maximal number of moles NADPH oxidized or oxidant reduced per mole of the enzyme active center per second, and *k*_cat_/*K*_m_, the bimolecular rate constant (or catalytic efficiency constant), corresponds to the inverse intercepts and slopes in Lineweaver-Burk coordinates, [E]/*v* vs. 1/[oxidant]. These rate constants were obtained by fitting the experimental data to the parabolic expression using the SigmaPlot 2000 (version 11.0, Systal Software, San Jose, CA, USA). The temperature dependence of reaction rate was examined at seven fixed temperatures between 15 and 45 °C in the absence of activators, the activation enthalpies and entropies of reaction were calculated from Eyring plots of ln (*k*_cat_/*K*_m_)/T vs. 1/T.

### 4.3. Cytotoxicity Assays

Murine hepatoma MH22a cells obtained from the Institute of Cytology of the Russian Academy of Sciences (St. Petersburg, Russia), were grown and maintained at 37 °C in DMEM medium, supplemented with 10% fetal bovine serum, 100 U/mL penicillin, and 0.1 mg/mL streptomycin, as described in [22]. In the cytotoxicity experiments, 3.0 × 10^4^/mL cells were seeded in 5-mL flasks either in the presence or in the absence of compounds and were grown for 24 h. In the absence of compounds, cells reached 40–50% confluence. The adherent cells were counted under a light microscope. Typically, they did not accumulate Trypan blue and their viability was 98.5–99.3%. Human colon adenocarcinoma cells HCT-116 obtained from ATCC (Manassas, VA, USA), were grown and maintained at 37 °C in 5% CO_2_ in RPMI 1640 DMEM medium, supplemented with 10% fetal bovine serum, 2 mM l-glutamine, and 0.05 mg/mL gentamycin. In the cytotoxicity experiments, 1.0 × 10^5^/mL cells were seeded in the absence or the presence of compounds and were grown for 48 h. In the absence of compounds, cells reached 65–75% confluence. Their viability was determined by staining with crystal violet [55]. Stock solutions of compounds were prepared in DMSO. Its concentration in cultivation media did not exceed 0.2% and did not affect cell viability. The experiments were conducted in triplicate.

### 4.4. Statistical Analysis and Calculations

The statistical analysis was performed using Statistica (version 4.3, Statsoft, Toronto, ON, Canada). Octanol/water distribution coefficients at pH 7.0 (log *D*) were calculated using LogD Predictor (https://chemaxon.com).

## Figures and Tables

**Table 2:** The steady-state rate constants of reduction of aromatic *N*-oxides by NQO1 and their calculated van der Waals volumes (VdWvol).

No.	Compound	*k*_cat_ (s^−1^)	*k*_cat_/*K*_m_ (M^−1.^s^−1^)	VdWvol (Å^3^) ^a^
1	7-CF_3_O-tirapazamine	0.11 ± 0.02	1.6 ± 0.1 × 10^3^	175.8
2	3-CH_3_CONH-1,2,4-benzotriazine-1,4-dioxide	3.40 ± 0.20	1.3 ± 0.1 × 10^4^	172.3
		3.00 ± 0.20 ^b^	1.0 ± 0.1 × 10^4 b^	
3	3-CH_3_OCONH-1,2,4-benzotriazine-1,4-dioxide	2.40 ± 0.22	3.0 ± 0.2 × 10^3^	180.7
4	3-CF_3_SO_2_NH-1,2,4-benzotriazine-1,4-dioxide	0.34 ± 0.06	1.0 ± 0.2 × 10^3^	203.5
5	3-CH_3_SO_2_NH-1,2,4-benzotriazine-1,4-dioxide	0.40 ± 0.05	1.2 ± 0.2 × 10^3^	184.9
6	2-CF_3_-quinoxaline-1,4-dioxide	13.5 ± 1.72	2.4 ± 0.2 × 10^4^	168.2
7	2-NH_2_-3-CN-quinoxaline-1,4-dioxide	0.11 ± 0.02	1.4 ± 0.1 × 10^3^	146.2
8	1,2,4-Benzotriazine-1-oxide	1.10 ± 0.12	2.1 ± 0.2 × 10^3^	111.7
9	3-CH_3_CONH-1,2,4-benzotriazine-1-oxide	0.06 ± 0.02	5.0 ± 1.0 × 10^2^	163.5
10	1,2,4-Benzotriazine-1,4-dioxide ^c^	1.00 ± 0.10	5.1 ± 0.7 × 10^3^	120.5
11	7-CF_3_-tirapazamine ^c^	1.30 ± 0.10	3.7 ± 0.4 × 10^3^	167.0
12	7-Cl-tirapazamine ^c^	1.00 ± 0.10	4.3 ± 0.4 × 10^3^	152.6
13	7-F-tirapazamine ^c^	0.70 ± 0.10	4.9 ± 0.6 × 10^3^	143.5
14	Tirapazamine ^c^	0.20 ± 0.10	2.4 ± 0.2 × 10^3^	131.5
15	7-CH_3_-tirapazamine ^c^	0.30 ± 0.05	1.7 ± 0.1 × 10^3^	148.8
16	7-C_2_H_5_O-tirapazamine ^c^	0.30 ± 0.04	1.0 ± 0.1 × 10^3^	174.9
17	3-Amino-1,2,4-benzotriazine-1-oxide ^c^	0.10 ± 0.02	1.6 ± 0.2 × 10^3^	122,7
18	Quinoxaline-1,4-dioxide ^c^	0.60 ± 0.10	7.9 ± 0.9 × 10^3^	126.8

^a^ Calculated according to [34], ^b^ Determined in the absence of activators, ^c^ Taken from Ref. [22].

**Table 3:** The logarithms of the geometric averages of ArN→O reactivity in P450R- and *Pf*FNR-catalyzed reactions (log *k*_cat_/*K*_m_ (avge)), their octanol/water distribution coefficients at pH 7.0 (log *D*), their concentrations for 50% cell survival (cL_50_) in murine hepatoma MH22a cells, and their concentrations causing 50% maximal proliferation inhibition (GI_50_) of human colon carcinoma HCT-116 cells.

No.	Compound	log *k*_cat_/*K*_m_ (avge)	log *D*	cL_50_ (µM) MH22a	GI_50_ (µM) HCT-116
1	7-CF_3_O-tirapazamine	4.62	1.32	3.6 ± 0.7	17 ± 3.0
2	3-CH_3_CONH-1,2,4-benzotriazine-1,4-dioxide	4.82	−0.82	1.5 ± 0.3	2.5 ± 0.5
3	3-CH_3_OCONH-1,2,4-benzotriazine-1,4-dioxide	4.78	−0.08	9.4 ± 1.4	6.3 ± 1.0
4	3-CF_3_SO_2_NH-1,2,4-benzotriazine-1,4-dioxide	4.41	−0.40	44 ± 6.5	125 ± 19
5	3-CH_3_SO_2_NH-1,2,4-benzotriazine-1,4-dioxide	3.67	−2.42	184 ± 25	185 ± 23
6	2-CF_3_-quinoxaline-1,4-dioxide	4.19	0.42	10 ± 2.0	12.5 ± 2.0
7	2-NH_2_-3-CN-quinoxaline-1,4-dioxide	3.96	−0.18	358 ± 52	125 ± 17
8	1,2,4-Benzotriazine-1-oxide	3.94	0.45	168 ± 21	225 ± 27
9	3-CH_3_CONH-1,2,4-benzotriazine-1-oxide	3.58	0.37	≥600	≥1000
10	1,2,4-Benzotriazine-1,4-dioxide	5.00	−0.70	11 ± 1.5 ^a^	18 ± 2.0
11	7-CF_3_-tirapazamine	4.83	0.76	3.4 ± 0.4 ^a^	6.0 ± 1.0 ^a^
12	7-Cl-tirapazamine	4.71	0.49	3.1 ± 0.5 ^a^	13 ± 1.5 ^a^
13	7-F-tirapazamine	4.48	0.03	7.2 ± 1.0 ^a^	25 ± 4.0
14	Tirapazamine	3.84	0.11	31 ± 5.5 ^a^ 28 ± 4.0	75 ± 7.0 ^a^ 64 ± 7.0
15	7-CH_3_-tirapazamine	3.82	0.40	83 ± 10 ^a^	50 ± 6.0 ^a^
16	7-C_2_H_5_O-tirapazamine	3.65	0.08	64 ± 10 ^a^	60 ± 7.0 ^a^
17	3-Amino-1,2,4-benzotriazine-1-oxide	3.48	0.30	≥600 ^a^	≥600 ^a^
18	Quinoxaline-1,4-dioxide	3.22	−0.90	325 ± 40 ^a^	≥800

^a^ Taken from Ref. [22].

**Table 4:** Modulation of the cytotoxicity of aromatic *N-*oxides in MH22a cells by desferrioxamine (DESF), *N,N′-*diphenyl-*p-*phenylenediamine (DPPD), 1,3-bis(2-chloroethyl)-1-nitrosourea (BCNU), and dicoumarol (DIC). The additions of compounds did not affect the cell viability in control experiments, 98.5–99.3%, *n* = 3, * *p* < 0.05, ** *p* < 0.02, *** *p* < 0.01.

No.	Compound	Cell Viability (%)				
		No Additions	Additions:			
			DESF (1.0 mM)	DPPD (2.5 µM)	BCNU (20 µM)	DIC (20 µM)
1	3-CH_3_CONH-1,2,4-benzotriazine-1,4-dioxide, 1.5 µM	47.2 ± 4.0	71.8 ± 4.0 ***	64.9 ± 3.8 ***	34.0 ± 3.0 **	83.7 ± 6.1 ***
2	2-NH_2_-3-CN-quinoxaline-1,4-dioxide, 350 µM	47.3 ± 4.2	83.5 ± 7.0 **	72.1 ± 5.7 **	35.3 ± 3.6 *	71.6 ± 5.1 **
3	1,2,4-Benzotriazine-1,4-dioxide, 10 µM	55.6 ± 4.5	n.d.	n.d.	n.d.	81.2 ± 7.4 **
4	3-CF_3_SO_2_NH-1,2,4-benzotriazine-1,4-dioxide, 40 µM	55.8 ± 4.9	n.d.	n.d.	n.d.	79.0 ± 3.1 **
5	2-CF_3_-quinoxaline-1,4-dioxide, 10 µM	57.6 ± 4.5	n.d.	n.d.	n.d.	83.1 ± 5.6 **

**Table 5:** The results of the multiparameter analysis of ArN→O cytotoxicity (Table 3) according to Equation (3).

Cell Line	*a*	*b*	*c*	*d*	*r* ^2^
	7.22 ± 0.80	−1.36 ± 0.19	-	-	0.7638
MH22a	7.07 ± 0.84	−1.33 ± 0.20	−0.09 ± 0.13	-	0.7704
	8.44 ± 0.88	−1.20 ± 0.18	-	−0.55 ± 0.24	0.8266
	8.30 ± 0.88	−1.14 ± 0.19	−0.13 ± 0.118	−0.59 ± 0.24	0.8405
	6.68 ± 0.73	−1.19 ± 0.17	-	-	0.7440
HCT-116	6.63 ± 0.79	−1.18 ± 0.19	−0.03 ± 0.13	-	0.7448
	7.74 ± 0.82	−1.05 ± 0.17	-	−0.48 ± 0.22	0.8052
	7.67 ± 0.85	−1.02 ± 0.18	−0.06 ± 0.11	−0.50 ± 0.23	0.8093

## Supplementary Materials

Supplementary materials can be found at https://www.mdpi.com/1422-0067/21/22/8754/s1.

## Abbreviations

ArN→O	Heteroaromatic *N-*oxide
BCNU	1,3-bis(2-chloroethyl)-1-nitrosourea
cL_50_	Concentration for 50% cell survival
DESF	Desferrioxamine
DIC	Dicoumarol
DPPD	*N,N*′-diphenyl-*p-*phenylene diamine
*E* ^1^_7_	Single-electron reduction midpoint potential at pH 7.0
GI_50_	Concentration for 50% inhibition of maximal cell proliferation
*k*_cat_	Catalytic constant
*k*_cat_/*K*_m_	Bimolecular rate constant
*k*_cat_/*K*_m_ (avge)	Geometric average of *k*_cat_/*K*_m_ in P-450R- and *Pf*FNR-catalyzed reactions
*k*_cat_/*K*_m_ (NQO1)	*k*_cat_/*K*_m_ in NQO1-catalyzed reaction
log *D*	Octanol/water distribution coefficient at pH 7.0
NQO1	NAD(P)H:quinone oxidoreductase
P-450R	NADPH:cytochrome P-450 reductase
*Pf*FNR	*Plasmodium falciparum* ferredoxin:NADP^+^ oxidoreductase
SOD	Superoxide dismutase
TPZ	Tirapazamine
