# Supplementary material for: Aerobic Cytotoxicity of Aromatic N-Oxides: The Role of NAD(P)H:Quinone Oxidoreductase (NQO1)
Source: Int J Mol Sci. 2020 Nov 19;21(22):8754. doi: 10.3390/ijms21228754 (PMC7699506; doi:10.3390/ijms21228754)
Supplement: Supplementary file 1 [file ijms-21-08754-s001.pdf]

International Journal of
Molecular Sciences

MDPI

Supplementary Information

Aerobic Cytotoxicity of Aromatic *N*-Oxides: The Role of NAD(P)H:Quinone Oxidoreductase (NQO1)

Aušra Nemeikaitė-Čėnienė [1], Jonas Šarlauskas [2], Lina Misevičienė [2], Audronė Marozienė [2], Violeta Jonušienė [3], Mindaugas Lesanavičius [2] and Narimantas Čėnas [2,*]

1 State Research Institute Center for Innovative Medicine, Santariškių St. 5, LT-08406 Vilnius, Lithuania; ausra.ceniene@imc.lt
2 Institute of Biochemistry of Vilnius University, Saulėtekio 7, LT-10257 Vilnius, Lithuania; jonas.sarlauskas@bchi.vu.lt (J.Š.); lina.miseviciene@bchi.vu.lt (L.M.); audrone.maroziene@bchi.vu.lt (A.M.); mindaugas.lesanavicius@gmail.com (M.L.)
3 Institute of Biosciences of Vilnius University, Saulėtekio 7, LT-10257 Vilnius, Lithuania; violeta.jonusiene@gf.vu.lt
* Correspondence: narimantas.cenas@bchi.vu.lt; Tel.: +370-5-223-4392

1. Materials and Methods

Bovine leukemia virus-transformed lamb embryo kidney fibroblasts (line FLK) obtained from Institute of Cytology of the Russian Academy of Sciences (St. Petersburg, Russia) were grown and maintained in Eagles medium supplemented with 10% fetal bovine serum and antibiotics as described in [1]. Murine embryonic liver cells (line BNL CL.2) obtained from ATCC (Manassas, VA, USA) were grown and maintained in Dulbecco's modified Eagle's medium (DMEM) supplemented with 10% fetal bovine serum and antibiotics as described in [2]. In the cytotoxicity experiments, 3.0×10^4/mL cells were seeded in 5-mL flasks either in the presence or in the absence of compounds, and were grown for 24 h. The adherent cells were counted under a light microscope. Typically, they did not accumulate Trypan blue and their viability was 97%–98%. Stock solutions of compounds were prepared in DMSO. Its concentration in cultivation media did not exceed 0.2%, and did not affect cell viability. The experiments were conducted in triplicate.

Primary mice splenocytes were obtained from 4- to 8-week old male and female BALB/c mice as described in [3]. These experiments were approved by the Lithuanian Veterinary and Food Service. After washing the cells twice with RPMI 1640 medium, they were resuspended at the concentration of 10^6 cells /ml in RPMI 1640 medium with 5% fetal bovine serum and antibiotics, and were used for the further experiments. Cell viability was determined after 24 h of incubation of splenocytes with the examined compounds in 96-well cell culture plates (200 μl suspension per well), according to a Trypan blue exclusion test.

For enzymatic analysis, FLK and BNL CL.2 cells were grown until confluency, detached by trypsinization, twice washed with 0.1 M K-phosphate, pH 7.0, containing 1.0 mM EDTA and 1.0 mM PMSF, and sonicated on ice in four cycles of 20 s. Typically, 1.0-1.5 × 10^6 cells were used. The homogenate was centrifuged at 14000 x g for 45 min and the resulting supernatant was used for enzymatic analysis. Protein amount was determined according to the method of Bradford. The kinetic measurements were carried out spectrophotometrically using a PerkinElmer Lambda 25 spectrophotometer (PerkinElmer, Waltham, MA, USA) in 0.1 M K-phosphate buffer (pH 7.0) containing 1 mM EDTA, 0.01% Tween 20 and 0.25 mg/mL bovine serum albumin at 37 °C. The activity of NAD(P)H:quinone oxidoreductase (NQO1) was determined following the rate of reduction of 50 μM cytochrome c ($\Delta\varepsilon_{550}$ = 20 $mM^{-1}\cdot cm^{-1}$) in the presence of 10 μM menadione and 150 μM NADPH, as the difference between the reduction rate in the absence of 20 μM dicoumarol, and in its presence.

2. Results

We found that the activity of NQO1 in FLK and BNL Cl.2 cells was equal to 240 ± 30 and 35 ± 4.0 nmol cytochrome *c* reduced × min^{-1} × mg $protein^{-1}$, respectively. The activity of NQO1 in primary mice splenocytes is equal to 4.0 ± 0.3 nmol cytochrome *c* × min^{-1} × mg $protein^{-1}$ [3]. The cL_{50} of several ArN→O representatives in above cell lines are given in Table S1.

Table S1. The compound concentrations for 50% cell survival (cL_{50}) in FLK, BNL CL.2 cells, and in primary mice splenocytes, 24 h, *n* = 3.

No.	Compound	cL_{50} (μM)		
		**FLK**	**BNL CL .2**	**Primary mice splenocytes**
1	3-CH_3CONH-1,2,4-benzotriazine-1,4-dioxide	33.0 ± 3.5	11.0 ± 1.0	3.8 ± 0.5
2	3-CH_3CONH-1,2,4-benzotriazine-1-oxide	85-90% viable cells in the presence of 1.0 mM compound	80-87% viable cells in the presence of 1.0 mM compound	n.d.
3	1,2,4-Benzotriazine-1,4-dioxide	n.d.	n.d.	5.3 ± 0.7
4	Tirapazamine	170 ± 7.0	160 ± 8.0	9.4 ± 1.0

The effects of dicoumarol on the cell viability in the presence of ArN→O are summarized in Table S2.

Table S2. The effects of 20 μM dicoumarol (DIC) on the cytotoxicity of ArN→O, 24 h, n = 3. This concentration of dicoumarol did not affect cell viability,

No.	Compound	Viable cells (%)		
		**FLK**	**BNL CL .2**	**Primary mice splenocytes**
1	3-CH_3CONH-1,2,4-benzotriazine-1,4-dioxide	[compound] = 30 μM:	[compound] = 11.0 μM:	[compound] = 4.0 μM:
		50.1 ± 3.8 (-DIC)<br>63.1±3.0 (+DIC)<br>$p < 0.05$	47.0 ± 3.4 (-DIC)<br>71.2 ± 4.5 (+DIC)<br>$p < 0.02$	50.0 ± 4.5 (-DIC)<br>48.0 ± 5.0 (+DIC)
2	1,2,4-Benzotriazine-1,4-dioxide	n.d.	n.d.	[compound] = 5.0 μM:
				52.5 ± 4.0 (-DIC)<br>55.3 ± 4.8 (-DIC)
3	Tirapazamine	[compound] = 170 μM:	[compound] = 160 μM:	[compound] = 10.0 μM:
		56.1 ± 3.0 (-DIC)<br>85.5±8.3 (+DIC)<br>$p < 0.001$	44.6 ± 4.1 (-DIC)<br>61.9 ± 4.0 (+DIC)<br>$p < 0.05$	47.5 ± 3.8 (-DIC)<br>50.2 ± 3.9 (-DIC)

References

1. Nemeikaitė, A.; Čėnas, N. The changes of prooxidant and antioxidant enzyme activities in bovine leukemia virus-transformed cells. Their influence on quinone cytotoxicity. *FEBS Lett.* **1993**, *326*, 65-68.
2. Liu, C.-H.; Chiu, T.-Y., Hu, M.-L. Fucoxanthin enhances HO-1 and NQO1 expression in murine hepatic BNL CL.2 cells through activation of the Nrf2/ARE system partially by its prooxidant activity. *J. Agric. Food Chem.* **2011**, *59*, 11344-11351.
3. Miliukienė, V.; Nivinskas, H.; Čėnas, N. Cytotoxicity of anticancer aziridinyl-substituted benzoquinones in primary mice splenocytes. *Acta Biochim. Pol.* **2014,** *61*, 833-836.
